# Adherence to and Efficacy of the Nutritional Intervention in Multimodal Prehabilitation in Colorectal and Esophageal Cancer Patients

**DOI:** 10.3390/nu15092133

**Published:** 2023-04-28

**Authors:** Sabien H. van Exter, Luuk D. Drager, Monique J. M. D. van Asseldonk, Dieuwke Strijker, Nina D. van der Schoot, Baukje van den Heuvel, Sjors Verlaan, Manon G. A. van den Berg

**Affiliations:** 1 Department of Gastro-Enterology and Hepatology, Dietetics and Intestinal Failure, Radboud University Medical Center, 6500 HB Nijmegen, The Netherlands; 2 Department of Operating Rooms, Radboud University Medical Center, 6500 HB Nijmegen, The Netherlands; 3 Department of Surgery, Radboud University Medical Center, 6500 HB Nijmegen, The Netherlands; 4 FrieslandCampina, 6708 WH Wageningen, The Netherlands; 5 Faculty of Sports & Nutrition, Amsterdam University of Applied Sciences, 1067 SM Amsterdam, The Netherlands

**Keywords:** prehabilitation, colorectal cancer, esophageal cancer, protein, nutrition, adherence

## Abstract

Multimodal prehabilitation programs to improve physical fitness before surgery often include nutritional interventions. This study evaluates the efficacy of and adherence to a nutritional intervention among colorectal and esophageal cancer patients undergoing the multimodal Fit4Surgery prehabilitation program. The intervention aims to achieve an intake of ≥1.5 g of protein/kg body weight (BW) per day through dietary advice and daily nutritional supplementation (30 g whey protein). This study shows 56.3% of patients met this goal after prehabilitation. Mean daily protein intake significantly increased from 1.20 ± 0.39 g/kg BW at baseline to 1.61 ± 0.41 g/kg BW after prehabilitation (*p* < 0.001), with the main increase during the evening snack. BW, BMI, 5-CST, and protein intake at baseline were associated with adherence to the nutritional intervention. These outcomes suggest that dietary counseling and protein supplementation can significantly improve protein intake in different patient groups undergoing a multimodal prehabilitation program.

## 1. Introduction

The cornerstone of most oncological treatment regimens is surgery. In colorectal (CRC) and esophageal cancer (EsC) surgery, postoperative complications occur in approximately 33% and 40% of patients, respectively [1,2]. These complications are associated with a prolonged hospital stay, increased mortality rate, hospital costs, and a lower reported quality of life [1,2,3,4,5]. Even in the absence of complications, both functional and physiological capacity are known to diminish in the postoperative period, as surgery represents a major stressor [6,7].

Preoperative physical fitness has consistently been associated with postoperative outcomes after major elective surgery [8,9,10,11]. Therefore, the preoperative period is increasingly considered an opportunity to improve patients’ preoperative physical status, a process termed prehabilitation. Modern prehabilitation programs encompass multiple modalities, including physical exercise, nutritional interventions, psychological support, and smoking cessation programs. Currently, multimodal prehabilitation has mainly been studied in CRC surgery, with patients showing increased preoperative physical fitness, reduced postoperative complications, and shortened length of stay [12,13,14].

Nutritional interventions within prehabilitation programs mainly focus on adequate protein, energy, and micronutrient intake. The average healthy person is estimated to require 0.83 g (g) of protein/kg body weight (BW)/day, while protein requirements for presurgical cancer patients are at least 1.2–1.5 g/kg BW/day [15,16,17]. However, many cancer patients do not meet this minimum recommended intake [18]. Compared to CRC patients, EsC patients are more often nutritionally compromised, which is the result of physical complaints such as rapidly developing dysphagia [19,20]. Since malnutrition has been associated with increased morbidity and mortality, early screening and optimization of the nutritional status in EsC patients awaiting surgery is common practice in the Netherlands. Dietary management in some EsC patients consists of enteral tube feeding or oral nutritional supplementation (ONS).

Patients participating in a prehabilitation program require a higher total intake of protein in order to stimulate exercise-induced muscle protein synthesis (MPS) [21]. Increased protein intake during prehabilitation programs is often achieved via providing patients with dietary advice and supplemental nutrients [22,23,24]. Protein intake is most effective for MPS when spread throughout the day, with multiple high-protein meals resulting in so-called protein peaks [25,26,27].

When striving for MPS, energy requirements should also be taken into account to maintain a stable or anabolic nutritional state [15]. Energy requirements can be calculated using the World Health Organization (WHO) formula, which includes a surcharge factor to account for physical activity or illness [28]. Complementary to this, micronutrients may be supplied to achieve the recommended daily intake, with a special focus on vitamin D, as this micronutrient plays an important role in maintaining muscle function and promotes protein synthesis [29,30].

Measuring adherence to a nutritional intervention is vital to determine the intervention’s efficacy and feasibility correctly. However, adherence to nutritional interventions in multimodal prehabilitation programs is often not reported or lacks a detailed description of nutritional intake [22,24,31,32]. This study aims to evaluate adherence to a nutritional objective of reaching a total protein intake of 1.5 g of protein/kg BW/day in CRC and EsC patients undergoing the multimodal Fit4Surgery prehabilitation program. Daily protein intake and distribution throughout the day are determined at baseline and after prehabilitation. Furthermore, energy intake and the intake of protein supplements and multivitamins are recorded. Additionally, it is investigated whether certain patient characteristics at baseline may be associated with nutritional adherence in order to further optimize the prehabilitation program based on specific patient characteristics and needs.

## 2. Materials and Methods

### 2.1. Study Design

In this exploratory prospective cohort study, data were derived from patients participating in the F4S PREHAB trial, performed in the Radboudumc (Nijmegen, The Netherlands). The trial received ethical approval from METC Oost-Nederland (NL73777.091.20). The trial has been registered in the International Clinical Trials Registry Platform (NL8699). The methods and outline of the study protocol can be found in the Appendices (Appendix A).

### 2.2. Study Population

Patients aged sixteen years and older, undergoing a multimodal prehabilitation program prior to elective high-impact surgery for CRC or EsC, were included.

Exclusion criteria in the F4S PREHAB trial were premorbid conditions (i.e., respiratory or cardiac disease) or impaired mobility that hampered or contraindicated exercise, cognitive disabilities, inability to read and understand the Dutch language, an American Society of Anesthesiologists (ASA) score ≥ 4 (a subjective assessment of a patient’s overall health), and chronic kidney disease at a stage ≥ 3. An additional exclusion criterion in this study was missing data regarding nutritional intake at baseline or after prehabilitation.

### 2.3. Study Outline

In addition to standard preoperative care, patients underwent a personalized multimodal prehabilitation program. The length of the program depended on the available time between diagnosis and surgery. In the case of neoadjuvant chemoradiotherapy, the start of the intervention was postponed until the last treatment. This multimodal prehabilitation program contained four different modalities: a nutritional intervention, an exercise program, psychological support, and a smoking cessation program (Appendix A).

Nutritional assessment occurred at baseline and prior to surgery (after prehabilitation), collecting the following parameters: BW, height, hand grip strength, fat-free mass measured through bioelectrical impedance analysis (BIA), and a three-day food diary. A general impression of a patient’s nutritional status was obtained using the Patient-Generated Subjective Global Assessment Short Form (PG-SGA SF). Additionally, all patients were referred to a registered in-hospital dietician in order to provide personalized dietary advice aiming for optimal nutritional intake (protein, energy, and micronutrients) to support achieving an anabolic state and enhancing the effect of physical training on lean body mass increment.

A daily protein intake of at least 1.5 g/kg BW was aimed for since it is fundamental for muscle health [33,34]. BW was corrected for patients with a body mass index (BMI) lower than 20 or higher than 30 to fit a BMI of 20 or 27.5, respectively [35,36]. Nutritional advice was given to aim for at least two meals per day containing 25 g of protein or more. Patients received high-quality whey protein shakes (Nutri Whey™ Isolate, FrieslandCampina, Wageningen, the Netherlands) containing 30 g of whey protein and 20 µg vitamin D. Patients were instructed to consume the protein shakes before bedtime on a daily basis, and an additional shake within one hour following supervised exercise. Patients relying on tube feeding received one sachet of PROSource NoCarb (Generic Life quality enhancing Niche Products, GLNP, Naarden, The Netherlands) daily, containing 15 g of protein. Energy requirements were calculated using the WHO formula with a 30% addition to account for physical activity. Additionally, daily multivitamin supplementation (50% of the daily recommendation) was provided to all patients to target possible vitamin deficiencies.

The exercise program was preceded by an assessment and screening using the American College of Sports Medicine (ACSM) exercise preparticipation health screening questionnaire to identify possible individuals at risk of exercise-related adverse cardiovascular events. Exercise was supervised by a first-line physiotherapist and performed two to three times a week, focusing on both resistance and high-intensity endurance training. Patients were also advised to perform 60 min of low-intensity aerobic exercise on days without supervised training.

Patients at risk for anxiety and depression were identified using the Hospital Anxiety and Depression Scale (HADS) and referred to a trained psychologist to improve coping mechanisms regarding future surgery. Furthermore, all active smokers were offered a smoking cessation program, including counseling and nicotine replacement therapy.

### 2.4. Study Outcomes

The primary aim of this study was to assess the adherence of patients to the nutritional intervention (daily protein intake ≥1.5 g/kg BW or ≥1.9 g/kg FFM [37]). The total protein intake was measured using three-day food records including two weekdays and one weekend day, at baseline and after prehabilitation, and expressed as g per day, g per kg BW per day, and g per kg fat-free mass (FFM) per day. Food records were structured according to meal moments. During dietary consultations at baseline and after prehabilitation, food records were reviewed by both patient and dietician to ensure an adequate description of dietary intake. A dietary history assessment was conducted in case of incomplete food records. Nutrient intake was calculated using Evry (version 2.7.4.2), a dietary calculation tool based on the Dutch Food Composition Table 7.0 (National Institute for Public Health and the Environment, Bilthoven, The Netherlands).

Secondary outcomes were protein distribution throughout the day (defined as the number of patients achieving ≥25 g in at least two meals per day), daily energy intake, and daily consumption of protein and multivitamin supplements. Furthermore, characteristics associated with adherence to the nutritional intervention were evaluated.

### 2.5. Statistical Analyses

Baseline characteristics of the study population and data regarding protocol adherence and nutritional intake were described separately for CRC and EsC patients. To determine potential associations with adherence, baseline characteristics were also described separately for adherent and non-adherent patients. Continuous data are presented as mean ± SD or median [inter-quartile range] in normally and non-normally distributed data, respectively, and compared using independent samples t-tests or Mann–Whitney U tests. Categorical data are described as total numbers (percentages) and compared using chi-square and Fisher’s exact tests. The difference in protein goal achievement between baseline and after prehabilitation was compared using McNemar’s test for related samples. Changes in protein intake per meal moment between baseline and after prehabilitation were compared using the Wilcoxon signed rank test.

## 3. Results

### 3.1. Baseline Characteristics of the Study Population

Between March 2021 and September 2022, 139 CRC and EsC patients were included in the F4S PREHAB trial. After the exclusion of 75 patients due to incomplete or missing data on nutritional intake at baseline or after prehabilitation, 35 CRC and 29 EsC patients were eligible for analysis (Table 1). Patients had a median age of 66 years at the time of inclusion and were predominantly male (70.3%), without significant differences between CRC and EsC patients. The PG-SGA SF score was significantly higher in EsC patients compared to CRC patients (7 [8] vs. 2 [4], *p* = 0.003). Ten (15.6%) patients were at high risk for malnutrition based on the PG-SGA SF score. Among EsC patients, 20 (69.0%) received either tube feeding or ONS at baseline.

### 3.2. Daily Protein Intake at Baseline and after Prehabilitation

**Figure 1:**
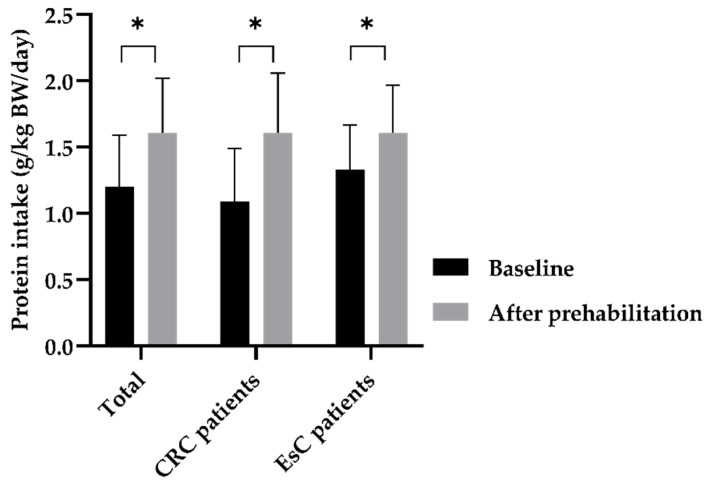
Daily protein intake corrected for body weight (BW) between baseline and after prehabilitation, presented for the total population (n = 64), CRC patients (n = 35), and EsC patients (n = 29). * *p* < 0.05.

Daily protein intake relative to kg BW increased significantly from 1.20 ± 0.39 g/kg BW at baseline to 1.61 ± 0.41 g/kg BW after prehabilitation in the total study population (*p* < 0.001). Analysis in CRC and EsC patients showed a significant increase in relative daily protein intake after prehabilitation compared to baseline, from 1.09 ± 0.40 g/kg BW and 1.33 ± 0.34 g/kg BW to 1.61 ± 0.45 g/kg BW and 1.61 ± 0.39 g/kg BW, respectively, with a higher increment in protein intake per kg BW in CRC patients (*p =* 0.006) (Figure 1).

**Figure 2:**
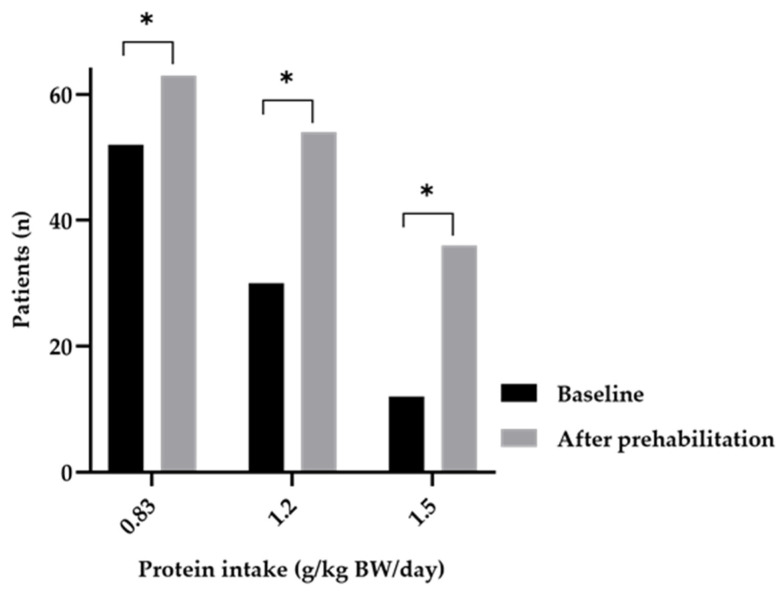
Number of patients achieving daily protein goals set to 0.83 g/kg BW, 1.2 g/kg BW, and 1.5 g/kg BW at baseline and after prehabilitation. * *p* < 0.05.

At baseline, the number of patients with a mean protein intake of ≥1.5 g/kg BW per day was 12 (18.8%), which increased to 36 (56.3%) following prehabilitation (*p* < 0.001) (Figure 2). Additional analyses comparing the number of patients achieving mean protein intakes of ≥1.2 and ≥0.83 g/kg BW at baseline and after prehabilitation showed increments of 30 (46.9%) to 54 (84.4%) patients and 52 (81.2%) to 63 (98.5%) patients, respectively, both statistically significant (*p* < 0.001). Considering the protein intake per kg FFM, a total of 46 (75.4%) patients achieved a mean protein intake of 1.9 g/kg FFM after prehabilitation, compared to 19 (31.1%) at baseline. Protein intake relative to FFM also significantly increased from 1.70 ± 0.66 g/kg FFM to 2.27 ± 0.75 g/kg FFM for the total study population (*p* < 0.001) (Appendix B).

The crude daily protein intake increased significantly in this study population from 93 ± 31 g/day at baseline to 124 ± 28 g/day after prehabilitation (*p* < 0.001). EsC patients had a significantly higher protein intake compared to CRC patients at baseline (107 ± 30 g/day vs. 82 ± 27 g/day, *p* < 0.001). However, there was no significant difference between the groups after prehabilitation (129 ± 30 g/day vs. 121 ± 27 g/day, *p =* 0.248). The total increase in protein intake was lower in EsC patients compared to CRC patients (22 ± 27 g/day vs. 39 ± 23 g/day, *p =* 0.009).

### 3.3. Daily Protein Intake at Baseline and after Prehabilitation per Meal Moment

**Figure 3:**
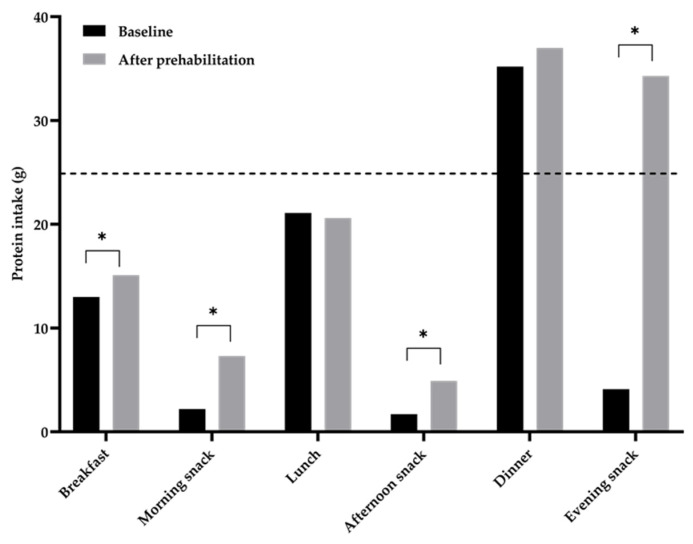
Median protein intake in grams (g) per meal for n = 54 patients at baseline and after prehabilitation. Threshold of 25 g protein visualized as dashed line. * *p* < 0.05.

Daily protein intake per meal moment was analyzed for 54 patients, since 10 patients received continuous tube feeding. Median protein intake was highest at dinner, both at baseline and after prehabilitation (Figure 3). Protein intake increased significantly during breakfast as well as morning, afternoon, and evening snacks, while the changes were non-significant for lunch and dinner. The largest increase in protein intake was during the evening snack (4.1 [7.5] g vs. 34.8 [8.3] g, *p* < 0.001).

A mean protein intake of ≥25 g in ≥2 meals per day was achieved by 43 (79.6%) patients, which was a significant increase when compared to 20 (37.0%) patients at baseline (*p* < 0.001). After prehabilitation, median protein intake was ≥25 g for dinner and the evening snack.

### 3.4. Energy and Supplement Intake

Mean (SD) energy intake at baseline was 2040 (570) kcal and significantly increased to 2287 (513) kcal after prehabilitation (*p* < 0.001). This meant that 29 (45.3%) patients achieved the energy requirement of WHO + 30% at baseline, and 46 (71.8%) achieved this after prehabilitation (*p* < 0.001).

Almost all patients (59 (92.2%)) reported daily consumption of protein shakes. Adherence to daily multivitamin supplementation was reported by 54 (84.4%) patients.

### 3.5. Characteristics Associated with Nutritional Adherence

There were no significant differences between adherence and non-adherence to the primary nutritional goal regarding age and sex. Patients who had a daily protein intake of ≥1.5 g/kg BW had a significantly lower BMI compared to patients who had not (25 [5] vs. 28 [4], *p* < 0.001). Patients who were adherent also had a faster 5-CST time in seconds (8 [3] vs. 9 [5], *p =* 0.017) and higher protein intake at baseline in g/kg BW/day (1.36 ± 0.39 vs. 0.99 ± 0.27, *p* < 0.001) compared to patients who were non-adherent (Appendix C).

## 4. Discussion

This study shows that 56% of patients with CRC and EsC met the primary criterion of ≥1.5 g protein/kg BW per day after prehabilitation. The total protein intake increased from 1.2 g/kg BW per day to more than 1.6 g/kg BW per day in this study population, without significant difference between CRC and EsC patients. Additionally, adherence to daily consumption of nutritional supplements was more than 90%. With a mean increment of 31 g of protein intake, supplementation seems essential in nutritional interventions as part of multimodal prehabilitation programs.

Protein intake per meal moment significantly increased during breakfast as well as morning, afternoon, and evening snacks. The distribution of protein intake across meal times was found to be skewed towards the end of the day. The largest increase in protein intake was observed during the evening snack, which corresponds to the protocol recommendation to consume daily protein shakes before bedtime. Consuming a high amount of protein during the evening can stimulate overnight muscle protein synthesis [38]. Although protein intake during breakfast increased significantly, it remained relatively low and could be improved through providing additional nutritional advice to achieve a high-protein breakfast. Protein supplementation at breakfast might be an effective way to increase protein intake during prehabilitation. Protein intake during lunch did not change significantly, but mean intake nearly reached the threshold of ≥25 g protein. Nutritional advice encouraging a small increase in protein during lunch could help reach the threshold of ≥25 g protein. Reaching a third or even fourth peak in protein intake would further stimulate MPS and remove the skewness of the protein intake distribution [39,40]. Our study found that nearly 80% of patients reached ≥2 mean peaks of ≥25 g protein per day. A previous study in preoperative patients prescribed six protein-rich dishes per day and reported ≥2 peaks of ≥20 g protein per day in merely one-third of patients [41]. This finding further emphasizes that protein supplementation can be helpful in attaining a high-protein diet in addition to dietary changes alone.

This study found several baseline characteristics that were associated with a lowered adherence to the primary goal of this nutritional intervention. A higher median BW and median BMI were seen in patients that did not achieve the protein goal. As protein goals were not adjusted to actual body composition, they may be more difficult to attain when BW increases. A technique to eliminate heightened protein goals in patients with a high BW due to fat mass instead of lean body mass is through using FFM measurements. The protein goal based on FFM is ≥1.9 g/kg FFM per day and is a more accurate way to determine protein requirements [42]. Results show 75% of patients achieved the protein goal based on FFM, which is higher than the 56% when protein requirements are based on BW. It is recommended that future trials use FFM to estimate protein requirements when measurements on FFM are available.

Patients that did not adhere to the daily protein goal showed a lower protein intake at baseline, both absolute and relative. This indicates that nutritional support may be even more important for patients with a lower protein intake at baseline. Intensive dietary counseling focusing on extra protein intake during breakfast and lunch may be helpful. Furthermore, patients with a reduced food intake may benefit from an additional serving of protein supplementation. Non-adherent patients also needed more time to complete the 5-CST at baseline. This suggests physical capacity at baseline may impact protocol adherence and possibly correlates with a prolonged protein deficit. Since protein intake is essential to retain and gain muscle strength, insufficient intake may lead to reduced muscle strength and physical performance [43].

To evaluate and compare nutritional adherence in patients with a compromised nutritional status, this study included both CRC and EsC patients undergoing elective surgery. EsC patients are often nutritionally compromised due to physical restrictions such as dysphagia and adverse effects caused by neoadjuvant treatment. However, these patients had higher protein intake at baseline. This could be the result of the majority of EsC patients (69%) receiving either tube feeding or ONS prior to prehabilitation. Furthermore, EsC patients already receive extensive dietary counseling before neoadjuvant treatment as part of standard preoperative care in the Netherlands. The higher protein intake at baseline in EsC patients supports the relevance of the early involvement of a registered dietician in the dietary management of those patients.

While some earlier prehabilitation studies did report adherence to the intake of nutritional supplementation, they did not address the total dietary intake before and after prehabilitation [44,45]. This study is one of the first to describe the assessment of adherence to a nutritional intervention in detail using validated methods. Furthermore, the results of this study show that a nutritional intervention as part of multimodal prehabilitation is feasible in different groups of patients. However, this study has some limitations. Firstly, the sample size of this study is relatively small, which limited statistical options to identify predictors regarding adherence. Secondly, selection bias might have been introduced in this study via the exclusion of patients based on missing data on food intake. It is plausible that patients who recorded their food intake at both timepoints also had higher overall motivation regarding the nutritional intervention as part of multimodal prehabilitation.

In conclusion, the nutritional intervention as part of multimodal prehabilitation increased protein intake significantly in both CRC and EsC patients during prehabilitation. This shows that dietary counseling and providing protein supplementation are effective methods to increase protein intake in cancer patients during prehabilitation. Further dietary counseling should be given to reach additional protein peaks during breakfast and lunch. Future research, preferably estimating protein requirements using FFM, should include larger study populations to identify predictors of protocol adherence more accurately. Qualitative research on the wishes and needs of patients regarding dietary advice and supplementation can help gain more understanding of how to optimize adherence to nutritional interventions.

## Figures and Tables

**Table 1:** Baseline characteristics of the study population.

Characteristic	All Patients (n = 64)	CRC (n = 35)	EsC (n = 29)	*p*-Value
Age in years, median [IQR]	66 [16]	65 [16]	66 [15]	0.746
Male sex (%)	45 (70.3%)	24 (68.6%)	21 (72.4%)	0.738
ASA score				
I	3 (4.7%)	3 (8.6%)	0 (0)	0.264
II	45 (70.3%)	24 (68.6%)	21 (72.4%)	
III	16 (25.0%)	8 (22.9%)	8 (27.6%)	
Weight in kg, median [IQR]	77 [19]	72 [26]	81 [12]	0.103
Fat free mass in kg, mean ± SD	54.5 ± 10.6	53.8 ± 11.3	55.4 ± 9.8	0.572
Fat-free mass index in kg/m^2^, mean ± SD	18.1 ± 2.3	18.1 ± 2.5	18.1 ± 2.1	0.954
BMI in kg/m^2^, median [IQR]	26 [4]	25 [8]	27 [2]	0.671
Smoking				0.357
No	19 (29.7%)	13 (37.1%)	6 (20.7%)	
Yes	8 (12.5%)	4 (11.4%)	4 (13.8%)	
Former smoker	37 (57.8%)	18 (51.4%)	19 (65.5%)	
Hand grip strength at baseline in kg, mean ± SD	38.0 ± 13.1	37.5 ± 14.4	38.6 ± 11.5	0.739
Male	43.5 ± 11.1	43.5 ± 13.0	43.6 ± 8.7	0.967
Female	25.1 ± 6.9	24.9 ± 7.6	25.4 ± 6.2	0.889
5-CST in seconds, median [IQR]	9 [4]	9 [4]	9 [5]	0.569
PG-SGA score, median [IQR]	3 [8]	2 [4]	7 [8]	0.003
PG-SGA categories (%)				<0.001
Low risk (≤3)	33 (51.6%)	25 (71.4%)	8 (27.6%)	
Moderate risk (4–8)	21 (32.8%)	8 (22.9%)	13 (44.8%)	
High risk (≥9)	10 (15.6%)	2 (5.7%)	8 (27.6%)	
Oral Nutritional Supplement (%)	10 (15.6%)	0 (0)	10 (34.5%)	<0.001
Tube feeding (%)	10 (15.6%)	0 (0)	10 (34.5%)	<0.001
Number of trainings, median [IQR]	9 [6]	10 [5]	8 [5]	0.267
Duration of prehabilitation in days [IQR]	34 [12]	33 [8]	35 [24]	0.204

## Data Availability

Data regarding this work are available from the corresponding author upon request.

## Appendix A. Study Protocol Methods and Outline

### Appendix A.1. Methods

#### Appendix A.1.1. Study Design

The Fit4Surgery PREHAB trial is a monocenter randomized stepped-wedge cluster trial in the Radboudumc in Nijmegen, the Netherlands. According to a stepped-wedge approach, a multimodal prehabilitation program will be implemented as standard preoperative care in one cluster every month. Patient inclusion and prospective data collection started in March 2021 and is planned to run for a total of 30 months. Additional data, regarding patients who underwent elective high-impact surgery in the two-year period prior to the start of this trial, will be gathered retrospectively.

#### Appendix A.1.2. Study Population

All patients aged sixteen years and older, undergoing elective high-impact surgery, will be included. Based on different diagnoses, patients will be grouped into twenty clusters: colon cancer, rectal cancer, esophageal cancer, liver cancer or metastases (of colorectal origin), pancreaticobiliary cancer, (retro)peritoneal malignancies, abdominal aortic aneurysm (open and endovascular repair), renal cancer, bladder cancer, supratentorial meningioma, hip arthrosis, hip or knee arthroplasty failure, head and neck cancer, mouth cancer, autologous breast reconstruction, ovarian cancer, endometrial cancer, and vulvar cancer.

Exclusion criteria are premorbid conditions (i.e., cardiac or respiratory disease) or impaired mobility that contraindicates or hampers exercise; cognitive disabilities; illiteracy (inability to read and understand the Dutch language); chronic kidney disease at stage ≥ 4, which contraindicates protein supplementation; and patients with an ASA score ≥ 4.

### Appendix A.2. Study Outline

#### Appendix A.2.1. Control and Intervention Group

Patients participating in the control group will undergo standard preoperative care according to Dutch guidelines. For study purposes and measurements, patients receive three additional appointments, combined with standard pre- and postoperative outpatient clinic visits. Data regarding physical fitness, nutritional status, mental health, and health behavior in the pre- and postoperative phase will be collected.

Patients participating in the intervention group will undergo a personalized multimodal prehabilitation program with a duration of three to four weeks, depending on the available time between diagnosis and surgery. The program contains four different elements: an exercise program, a nutritional intervention, psychological support, and smoking cessation. Outcomes regarding these elements will be gathered in advance of the prehabilitation program and shortly before surgery (after prehabilitation). Prior to the start of the program, hemoglobin and glucose levels will be determined and will be addressed and treated accordingly.

#### Appendix A.2.2. Exercise Program

The content of the exercise programs consists of:

- Endurance training (HIIT):
  - Interval training with a total duration of 28 min, consisting of a 2 min warm-up, followed by alternating intervals of high intensity (4 intervals of 4 min) and moderate intensity (4 intervals of 3 min).
  - High-intensity workload is dosed at 90% of the peak wattage obtained via the steep ramp test (corresponding to an estimated 90% VO_2_ peak) and aims to reach Borg scores of 15–17 and ≥85% of the age-predicted maximal heart rate [46,47].
  - Moderate intensity workload is dosed at 30% of the peak wattage.
  - Examples of aerobic exercise machines on which HIIT may be performed are: bicycle, rower, treadmill, and cross-trainer.
  - Workload should be adjusted by 5–10% when patient is not able to complete the high intensity intervals.
- Resistance training:
  - Training targeting all major muscle groups composes six exercises (leg press, chest press, abdominal crunch, low row, lat pulldown, and step up) and consists of two series of 10 repetitions.
  - The strength exercises are performed according to two seconds of concentric strength and 2 s of eccentric strength. The weight of the exercises will be adjusted to the muscle strength (indirect 1RM) measured at baseline using the Brzycki formula (1RM = weight/(1.0278–0.0278 ◦ number of repetitions)) [48]. Exercises start at 65% of the calculated 1RM with a weekly increase of 5%, resulting in 80% of baseline 1RM in the fourth exercise week.
  - Weight should be adjusted by 5–10% according to a patients’ ability to perform 10 repetitions in the second series.
- Unsupervised training:
  - Patients are instructed to perform at least 60 min of aerobic exercise on days without supervised training. This may be divided into 2–3 periods of 20–30 min in case of insufficient physical capacity. Examples of aerobic exercises are: walking, cycling, and swimming.
  - All patients will be given advice regarding adequate rest and sleep.

#### Appendix A.2.3. Nutritional Intervention

The content of this modality is described in Section 2.3.

#### Appendix A.2.4. Psychological Support

Planned surgical treatment can be accompanied by anxiety and depression. These components could have a potential influence on motivation regarding prehabilitation and postoperative outcomes. Therefore, patients at risk will be identified using the Hospital Anxiety and Depression Scale (HADS).

Patients at risk will be referred to a trained psychologist to optimize psychological well-being and learn about coping mechanisms regarding the surgical treatment. Additional sessions will be planned in the preoperative period when necessary.

#### Appendix A.2.5. Smoking Cessation Program

All patients who are active smokers during the baseline assessment will be offered a smoking cessation program including counseling and nicotine replacement therapy prior to surgery. With a substantial number of patients being smokers at the time of (cancer) diagnosis, the goal is to achieve a smoking cessation rate of 80%.

## Appendix B. Protein Intake per kg FFM

**Table A1:** Number of participants achieving a goal intake of 1.9 g of protein/kg FFM. Presented for the total population (n = 61), CRC patients (n = 33), and EsC patients (n = 28).

	Baseline	After Prehabilitation
Total (n = 61)	19 (31.1%)	46 (75.4%)
CRC (n = 33)	6 (18.1%)	23 (69.7%)
EsC (n = 28)	13 (46.4%)	23 (82.1%)

**Table A2:** Daily protein intake relative to FFM: median [IQR]. Presented for the total population (n = 61), CRC patients (n = 33), and EsC patients (n = 28).

	Baseline	After Prehabilitation	*p*-Value
Total (n = 61)	1.70 [0.66]	2.27 [0.75]	<0.001
CRC (n = 33)	1.45 [0.68]	2.27 [0.85]	<0.001
EsC (n = 28)	1.80 [0.86]	2.27 [0.61]	0.001

## Appendix C. Baseline Characteristics of Adherent and Non-Adherent Patients

**Table A3:** Characteristics based on adherence to protein intake of ≥1.5 g (g)/kg BW (body weight)/day.

Characteristic	All Patients (n = 64)	Adherent (n = 36)	Non-Adherent (n = 28)	*p*-Value
Age in years, median [IQR]	66 [16]	65 [16]	67 [14]	0.386
Male sex (%)	45 (70.3%)	28 (77.8%)	17 (60.7%)	0.173
ASA score				0.763
I	3 (4.7%)	2 (5.6%)	1 (3.6%)	
II	45 (70.3%)	24 (66.7%)	21 (75.0%)	
III	16 (25.0%)	10 (27.8%)	6 (21.4%)	
Weight in kg, median [IQR]	77 [19]	72 [20]	81 [23]	0.003
Fat free mass in kg, mean ± SD	54.5 ± 10.6	53.7 ± 9.3	55.6 ± 12.1	0.503
Fat-free mass index in kg/m^2^, mean ± SD	18.1 ± 2.3	17.8 ± 2.0	18.6 ± 2.5	0.155
BMI in kg/m^2^, median [IQR]	26 [4]	25 [5]	28 [4]	<0.001
Smoking				0.145
No	19 (29.7%)	9 (25.0%)	10 (35.7%)	
Yes	8 (12.5%)	7 (19.4%)	1 (3.6%)	
Former smoker	37 (57.8%)	20 (55.6%)	17 (60.7%)	
Hand grip strength at baseline in kg, mean ± SD	38.0 ± 13.1	38.1 ± 12.1	37.9 ± 14.5	0.961
5-CST in seconds, median [IQR]	9 [4]	8 [3]	9 [5]	0.017
PG-SGA score, median [IQR]	3 [8]	3 [8]	4 [8]	0.732
PG-SGA categories (%)				0.513
Low risk (≤3)	33 (51.6%)	20 (55.6%)	13 (46.4%)	
Moderate risk (4–8)	21 (32.8%)	12 (33.3%)	9 (32.1%)	
High risk (≥9)	10 (15.6%)	4 (11.1%)	6 (21.4%)	
Type of cancer				0.454
CRC	35 (54.7%)	18 (50.0%)	17 (60.7%)	
EsC	29 (45.3%)	18 (50.0%)	11 (39.3%)	
Protein intake at baseline				
g/day mean ± SD	93.1 ± 30.7	101.1 ± 34.1	82.8 ± 22.2	0.017
g/kg BW/day ± SD	1.20 ± 0.39	1.36 ± 0.39	0.99 ± 0.27	<0.001
Number of trainings, median [IQR]	9 [6]	8 [5]	9 [7]	0.523
Duration of prehabilitation in days [IQR]	34 [12]	35 [24]	34 [12]	0.473
